# Effects of mindfulness on test anxiety: a meta-analysis

**DOI:** 10.3389/fpsyg.2024.1401467

**Published:** 2024-06-24

**Authors:** Eda Yılmazer, Zeynep Hamamci, Fulya Türk

**Affiliations:** ^1^Department of Psychology, Beykoz University, Istanbul, Türkiye; ^2^Department of Psychology, Yildiz Technical University, Istanbul, Türkiye

**Keywords:** mindfulness-based interventions (MBIs), test anxiety, meta-analysis, anxiety, school environment

## Abstract

**Objective:**

This meta-analysis evaluated the effectiveness of mindfulness-based interventions (MBIs) on test anxiety across diverse age groups and intervention modalities.

**Methods:**

Rigorous inclusion criteria were applied to select studies focusing on MBIs as the independent variable, with test anxiety as the outcome. A comprehensive search across multiple databases yielded 18 primary studies, contributing 20 comparisons. Data were extracted on study characteristics, sample sizes, and intervention details, and were analyzed using a random-effects model.

**Results:**

The analyses incorporated 1,275 participants, with MBIs demonstrating a moderate to large negative effect on test anxiety (effect size = −0.716; 95% CI: −1.383 to −0.049). Moderation analysis indicated that mean age, number of sessions, and intervention delivery mode did not significantly influence effect sizes. Publication bias assessment suggested the presence of bias via Egger’s regression (*p* = 0.025), though Begg and Mazumdar’s test and Duval and Tweedie’s trim and fill method indicated no missing studies.

**Conclusion:**

MBIs are effective in reducing test anxiety, though results should be interpreted with caution due to potential publication bias and unexplained heterogeneity. The impact of MBIs did not vary significantly with participant age, number of sessions, or delivery mode.

## Introduction

Exams, which are difficult for students of all ages, cause performance anxiety. It is an important problem in many countries that can affect students’ achievement and academic performance (D’Agostino et al., 2022). Intense test anxiety causes students to feel emotionally and cognitively challenged and helpless and ultimately negatively affects test success. Test anxiety has become a serious problem as it causes many different negative consequences such as low self-esteem, dropping out of school, lack of social skills, performance anxiety, academic failure and the emergence of various psychological problems (Hughes, 2005). On average, one in two students in Organization for Economic Cooperation and Development (OECD) countries worry about the difficulty of exams and feel very anxious even if they are well prepared for the exam, and school-related anxiety is negatively associated with school performance and life satisfaction (OECD, 2017).

Test anxiety has been defined as the emotional, physiological, and behavioral reactions surrounding the possible consequences of negative evaluation in an upcoming test or exam (Zeidner, 1998). Test anxiety has two main sources: anxiety and affectivity. While the anxiety dimension mostly includes cognitive concerns related to one’s performance, affectivity is mostly autonomous reactions to the test situation (Hembree, 1988). Von der Embse et al. (2018) conducted a meta-analysis study on test anxiety for more than 30 years and found that standardized tests, university entrance exams, grade point average, perceived test difficulty, avoidant coping behaviors, achievement goals, age, gender, self-esteem and self-efficacy were related to test anxiety (Von der Embse et al., 2018).

It is generally assumed that the presence of evaluation stress increases state anxiety, especially in individuals with high trait anxiety. People with high trait anxiety are said to be more sensitive to evaluation anxiety. People with high trait anxiety are characterized by their predisposition to perceive threats. According to Eysenck’s Attentional Control Theory, anxiety disrupts the effective functioning of the goal-directed attention system, especially the two executive functions (inhibition and shifting). When anxious thoughts utilize the limited resources of working memory, fewer resources may remain for targeted tasks (Eysenck et al., 2007). As individuals become more aware of where their attention is, they begin to notice the sensations, thoughts and feelings that arise within them. When we realize that we have been distracted or given full attention to a thought, we can slowly return awareness back to the original object of focus and then watch where the mind goes next. Mindfulness can be defined as “noticing the present experience with acceptance” (Linehan, 1993).

Mindfulness is an awareness of thoughts, feelings and bodily sensations. Mindfulness requires directing and accepting attention to what is happening in the present moment without judgment (Bishop et al., 2004). Although its positive effects are known in many psychological problems, it contributes to the success of individuals by reducing their test anxiety to a tolerable level. Many scientific studies have been conducted on Mindfulness based interventions (MBI) (Kabat-Zinn, 2003; Rapgay and Bystrisky, 2009; Malinowski, 2013). Mindfulness-based programs aim for individuals to learn this approach and use it in their daily lives. Mindfulness-based interventions help individuals to direct their attention to the here and now and learn to approach thoughts differently by realizing that thoughts are just thoughts (Bishop et al., 2004). Mindfulness interventions teach individuals mindfulness-based skills to use more functional strategies by accepting negative thoughts and emotions as they come to mind without judgment (Segal et al., 2012) Individuals increase their awareness through various exercises and practices and learn not to be captive to the negative thoughts that come to their minds. Through mindfulness practices, some cognitive processes such as cognitive flexibility and attention improve (Kuyken et al., 2010).

When we are consciously aware, we experience the world directly, not just through the lens of thought. Thoughts are not reality itself but just symbols and representations that symbolize reality. Mindfulness is in many ways a simple skill, because it only requires using the five senses to become aware of what is happening in the moment (Cardaciotto et al., 2008). While it is easy to be consciously aware for a minute or two, it is difficult to maintain this state of mind. This is against other natural tendencies of the brain. The brain uses its free time to focus on possible problems that need to be solved. This is evolutionarily useful, as it allows us to anticipate threats to our survival. But it is a dysfunctional way to live. Mindfulness is the resource that gives people the security they need, to meet challenging experiences with less resistance. Stress is part of everyday life. In the event of stress, thoughts, emotions and the body are affected and reflected in behavior. Stress distracts attention and concentration, increases anxiety and weakens social skills (Bartlett et al., 2021). Thus, when problems arise, people can act calmly, predictably and get more efficient results. Attention is an important mental power. Through mindfulness techniques the mind trains its attention, can pay more attention to what it wants to achieve and increase performance, it can give more constructive solutions instead of stress and anxiety (Maran et al., 2021).

By training the mind people can bring their attention to daily movements and to the “now” and the activities they are working on. All mindfulness practices draw attention to the present moment. The breath or any other object of focus for mindfulness is always in the present moment. Everything happens in the present moment. When attention is hijacked by a strong sensation or emotion, people lose the present moment. This does not mean that the present moment is really lost; it means that the experience of the present moment is lost. When attention is strong, people can do what they do moment by moment (Purser and Loy, 2013).

Studies have shown that mindfulness approach is effective in regulating students’ anxiety states. Studies have revealed that it affects the density of gray matter in the brain, increases the activation of the prefrontal cortex and reduces the activation of the amygdala, which is activated in situations such as fear and anxiety. Mindfulness interventions leads to permanent and positive changes in anxiety-related areas of the brain (Lutz et al., 2008a; Chiesa and Serretti, 2010; Goldin and Gross, 2010; Hölzel et al., 2011; Taren et al., 2013; Hernández-Saca, 2016). It has been revealed that the amygdala is less activated when individuals add mindfulness practices to their daily practices (Lutz et al., 2008b; Goldin and Gross, 2010). Researchers believe that “the act of deliberately activating the relaxation response inhibits the activation of the sympathetic nervous system in favor of the parasympathetic nervous system” (Edenfield and Saeed, 2012). During mindfulness meditation, the parasympathetic nervous system is activated (Edenfield and Saeed, 2012). And researchers stated that the anxiety response and the relaxation response cannot coexist at the same time. In summary, when the severity of test anxiety increases and its duration is prolonged, deterioration in the performance and disfunctionality in individual’s lives occurs (Linsay, 2002). One of the methods that can be applied to reduce test anxiety to the desired level is mindfulness intervention techniques. If children and adolescents start to benefit from mindfulness practices, they will be more competent in situations such as life stress, academic evaluation, performance anxiety and exam anxiety that they will face in the future. Mindfulness is called an approach that is a mixture of perception and acceptance of what is perceived; mindfulness is to perceive the present moment with a conscious mind and an open, loving and compassionate heart (Kiken et al., 2017).

In previous studies, there are meta-analysis studies showing that mindfulness interventions are effective in reducing anxiety levels (Bamber and Morpeth, 2019; Breedvelt et al., 2019). These meta-analysis studies examined the effect on general anxiety levels. In meta-analysis studies examining the effect of previous test anxiety interventions, they emphasized that the results of techniques including behavioral therapy, cognitive-behavioral therapy and mixed approaches were positive (Ergene, 2003; Von der Embse et al., 2013). However, it is seen that mindfulness-based interventions are quite limited in these studies. It is thought that this study will provide guidance for future research and contribute to the comparison of different interventions. Therefore, the aim of the study is to examine the effect of mindfulness-based interventions on test anxiety. It was also examined whether the effects of mindfulness-based interventions varied depending on age, number of sessions, session frequency and intervention delivery format (online or face-to-face).

## Methods

### Inclusion/exclusion criteria

#### Inclusion criteria

In line with the aim of the research, the researches obtained from the literature review were evaluated within the framework of the following criteria:

- Being a research written in English language.
- Being a research conducted using an experimental design.
- Conducting the study with students at all levels of education.
- The intervention to the level of test anxiety in the study is based on mindfulness-based approach.
- One of the dependent variables of the study is the test anxiety levels of individuals.
- Including the arithmetic mean and standard deviation values of individuals’ test anxiety levels and the number of participants.

According to the criteria given in the research, firstly, non-English studies were eliminated. Then, non-experimental studies, studies in which the study group was not students, studies conducted with other theoretical orientations, studies that did not include a control group or studies with different dependent variables other than test anxiety were also excluded. As a result of this elimination process, two articles were excluded from the study due to the lack of standard deviation values in two articles and also the control group was not included in the other four studies. For this reason, it was decided to use articles and thesis that met these criteria in the study and 18 studies that met the criteria were identified.

### Data selection

In this meta-analysis, we wanted to combine the results of studies that examined the exam anxiety of mindfulness studies in a nonclinical population.

We searched for publications in the major psychological databases Proquest, Science Direct, Springer link, Taylor & Francis, Web of science, Wiley with the descriptors, “mindfulness,” mindfulness studies, exam- test anxiety, mindful breathing, mindful drawing, brief mindful intervention” In addition, we scanned the references and citations of articles, reviews, and meta-analyses. When we found references of dissertations, we additionally checked if they had been published in the meantime. We included all studies that had been published by 2007–2022.

To be included, primary study researchers must have used mindfulness-based interventions with students and measured specifically test anxiety as an outcome. Both undergraduate, graduate, secondary school, college students were included. We included studies of non-clinical student samples (investigator measured). To be included, studies had to be written in English.

We included primary studies with two-group comparisons (MBI vs. control) as well as studies with pre-test/post-test analysis of MBI (one-group MBI). For the two-group comparisons, we included studies with an MBI group and a no-treatment control group. For the pretest/posttest comparison, we included studies where researchers employed single group pre- test/post-test designs.

In this meta-analysis, we adhered to specific inclusion criteria to ensure the rigor and relevance of our investigation. The studies selected for analysis were required to utilize mindfulness-based interventions (MBIs) as the primary independent variable, with a specific focus on test anxiety as the outcome variable. Our analysis was restricted to experimental study designs; consequently, correlational and qualitative studies were excluded. Eligible experimental studies needed to include both pre- and post-test comparisons, as well as a control group. There were no restrictions based on age group; studies encompassing diverse age demographics were included. Language and publication criteria were also established: studies had to be conducted in English and either published in an academic journal or accepted as a master’s or doctoral thesis. Lastly, it was imperative for the studies to provide necessary statistical data, including means, standard deviations, and sample sizes (N) for both experimental and control groups, to facilitate a comprehensive statistical analysis.

### Search strategy

Through search conducted in multiple electronic databases up until May 2023, we sought to identify relevant primary studies. The databases consulted included Seven electronic databases were searched for the studies to be included in the study. These are; Proquest, Science Direct, Springer link, Taylor&Francis, Web of Science, Wiley.

Key search terms employed were “mindful” in conjunction with “test anxiety.” The use of an asterisk as a truncation symbol enabled the inclusion of all possible word endings. Furthermore, to maximize the comprehensiveness of the search, subject headings were thoroughly expanded. A follow-up systematic search was executed employing the same methodology to capture any new studies that may have been published or overlooked during the initial search process, thereby ensuring a comprehensive inclusion of relevant literature in our analysis.

### Data extraction

To facilitate structured data collection and analysis, a codebook was developed. It included the following components: study reference (author’s last name and publication year), intervention duration (specified as one session, several weeks, etc.), the number of intervention sessions (counting each meeting as a session), mode of intervention delivery (online or face-to-face), utilized measurement tools along with their respective scoring range (minimum and maximum scores), mean age of participants, and for both experimental and control groups, the sample size, pre-test and post-test means and standard deviations. Additional data extracted included the length of each session, the intervention’s domain, and the type of source (academic article or thesis).

### Coding process

The coding process used in the research consists of three parts. In the first part, the imprint of the studies is given. In this context, study number, author/authors, year of publication, type of publication are given. In the second section, information about the content of the research is given. In this context, the name of the mindfulness intervention or technique applied in the research, the application period, the name of the scale applied, the number of sessions, the follow-up test, the placebo group, the session duration and the type of application are given. In the last section, coding was made by giving information about the research data, namely the numbers, averages and standard deviation values of the research groups. In the data coding phase, firstly, all studies were filed in a folder in the form of an electronic file with PDF extension. Then, each study was listed in Microsoft Excel worksheet according to author names and study number. A command connection was established between author names and PDF files. Thus, the necessary connection was provided to obtain the desired information during the coding process. In the third part of the coding process, the coding process was completed by including the data related to the research groups, mean and standard deviation values together with the study numbers and author names of the studies on a separate research Microsoft Excel worksheet.

In all of the studies whose effects were analyzed in this study, different scales were used to measure test anxiety. Sarason test anxiety, Revised test anxiety, State trait anxiety, and Test anxiety scale for maths were used to measure pre-test anxiety level.

### Data analyses

Data entry was performed in duplicate to ensure accuracy, and any inconsistencies were rectified. Effect sizes for comparisons between treatment and control groups at the conclusion of the interventions were computed using RStudio software (version 2023.12.0 + 369). We employed a random-effects model that postulates variation in true effect sizes across studies, attributable to disparities in participant demographics and intervention protocols. This approach presupposes a normal distribution of the true effect sizes. Each study was allocated a weight inversely proportional to its internal and between-study variance, facilitating the calculation of the aggregate effect size. To adjust for potential small sample size distortions, Hedge’s g values were calculated. Confidence intervals were set at 95%, assuming a standard normal distribution while accommodating a 2.5% margin of error on both extremes. We assessed heterogeneity by reviewing Forest plots and computing the Q statistic for overall variance, T2 for inter-study variance, and I2 to measure the proportion of effect size variability due to genuine differences among studies. Moderator analyses incorporated variables such as participant age, mode of intervention (online versus face-to-face), session count, and intervention length to investigate sources of heterogeneity. For categorical moderators, we applied methods analogous to ANOVA, whereas for continuous variables, we conducted meta-regression akin to multiple regression. Publication bias risk was evaluated through various methods, including funnel plot analysis of standard errors, Egger’s regression, the Begg and Mazumdar rank correlation test, and the Duval and Tweedie trim and fill procedure. This last method entails estimating and adjusting for the number of missing studies that might skew the funnel plot, recalculating the central effect size and variance accordingly. A significant reduction in the overall effect size raised concerns about potential publication bias. MS Office Excel 2007 and Comprehensive Meta-Analysis (CMA) program were used to create the calculations, tables and graphs used in the findings and interpretation section of the study.

## Results

**Figure 1:**
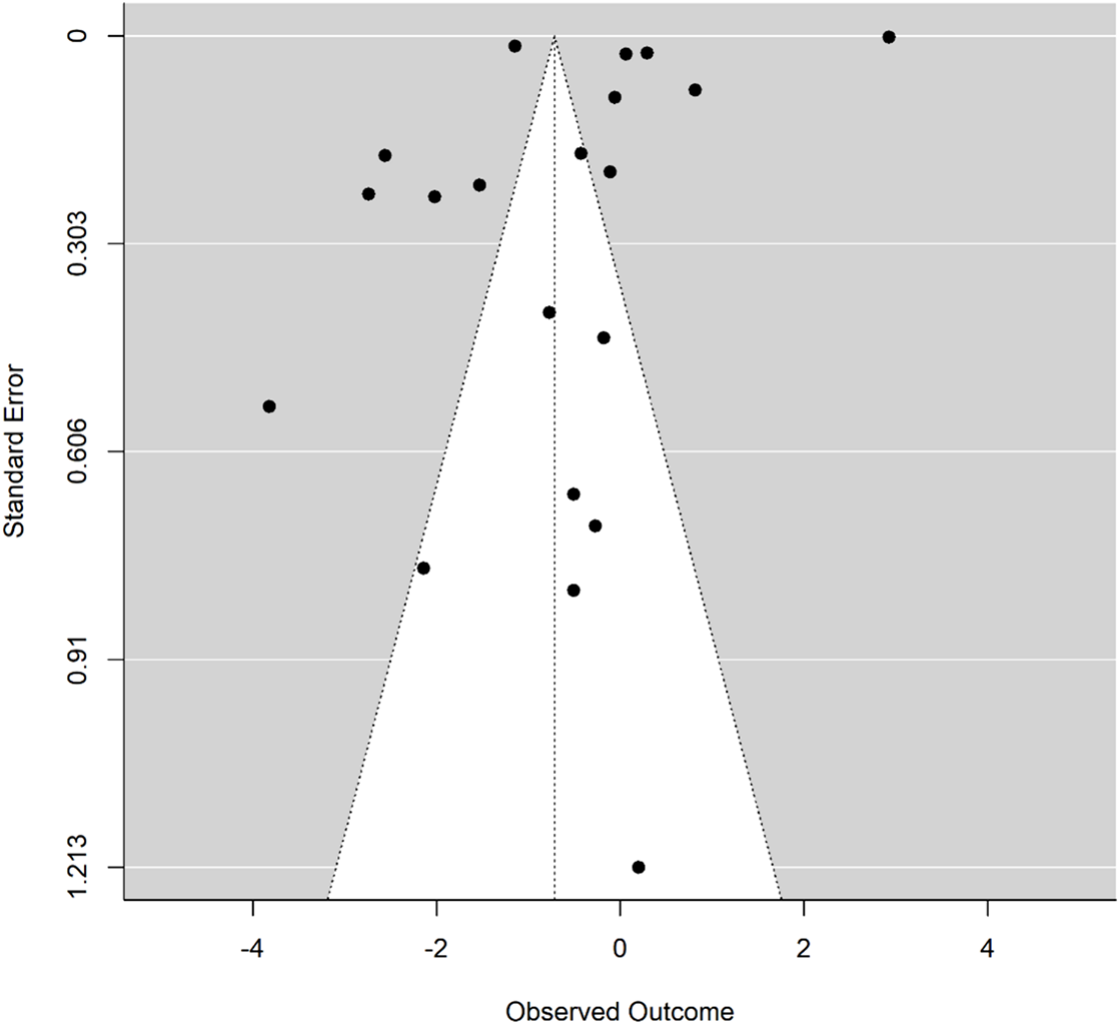
Funnel plot observed effect sizes (Cohen’s d) against standard errors.

A meticulous search strategy was executed across multiple databases to collate studies investigating the impact of mindfulness-based interventions (MBIs) on test anxiety. Our search encompassed articles up to year 2022, ensuring a comprehensive capture of relevant research. The initial retrieval from electronic databases yielded a substantial volume of studies, which was then meticulously refined. Duplicates were systematically removed, leaving 86 studies for closer examination. The first screening stage involved assessing abstracts against our stringent inclusion criteria, which focused on the use of MBIs as an independent variable and test anxiety as an outcome measure. This stage concluded with 27 studies earmarked for full-text review. Each potential study underwent a thorough full-text evaluation to confirm compliance with our inclusion standards. During this scrutiny, studies lacking adequate statistical information, such as pre-test and post-test means and standard deviations, were excluded. Despite efforts to retrieve missing data by contacting authors, some studies were inevitably omitted from further analysis due to incomplete datasets. Of particular note, one study presented two distinct interventions—one delivered online and the other face-to-face both compared against a control group. To account for the unique characteristics and potential differential effects of each intervention mode, we considered these as separate comparisons within our analysis. Another study explored the efficacy of MBIs over varying durations, specifically 3 weeks and 8 weeks, also in comparison to a control group. Given the differential time frames, these were likewise treated as individual studies. Our meticulous selection process culminated in the inclusion of 18 studies, which encapsulated 20 comparisons due to the distinct intervention types and durations within certain studies. This rich dataset provided a robust foundation for our meta-analytical investigation into the effectiveness of MBIs on test anxiety (see Figure 1).

### Descriptive statistics

The analysis encompassed 18 primary studies, yielding 20 distinct comparisons. The studies included a cumulative total of 1,275 participants, with 595 enrolled in mindfulness-based intervention (MBI) groups (mean = 29.75, SD = 21.61) and 680 in control groups receiving no treatment (mean = 34.00, SD = 28.23). Geographically, the studies were predominantly conducted in the United States (9 studies), followed by Iran (4 studies), and one each in Canada, India, Iraq, Norway, and South Korea. The majority of the studies (15) were journal publications, while the remaining three were doctoral dissertations.

The average age of participants across studies was 18.93 years, with a standard deviation of 4.52. The duration of MBI programs averaged 4.05 weeks, with a standard deviation of 3.03, encompassing an average of 9.2 sessions (SD = 11.43). Out of the 20 comparisons, 15 were conducted face-to-face, and 5 employed online modalities. Descriptive characteristics of these studies are given in Table 1.

**Table 1:** Main characteristics of studies included in the meta-analysis.

Study (country)	Year	Experimental gruoup N	Grade levels	Intervention (n sessions, duration in weeks)	Control group N	Intervention	Follow-up time	Inventory used
Zandi et al., Iran	2021	20	Secondary high school	8 sessions training based on mindfulness	20	No intervention	2 weeks	Sarason test anxiety scale
Dundas et al., Norway	2016	46	Bachelor, master’s and college students	8-week course designed for the MBSR program, and consisted of eight meetings and a day-long meditation class between meetings six and seven/four licensed clinical psychologists led the five groups, working in teams of two therapists (for three of the groups) or as a single therapist (for two groups). Each group had at least one therapist with a regular and long-term personal mindfulness practice who had attended formal MBSR training, including the “teacher intensive” training offered at the Center for Mindfulness at the University of Massachusetts.	90	No intervention	6 months, 1 year, 1.5 years, 2 years, and 2.5 years after the intervention	Revised test anxiety scale
Sohrabi et al., Iran	2013	49	High school	Mindfulness education	49	No intervention	No follow-up mentioned	Spilburger anxiety test
Priebe and Costes, USA	2022	45	Undergraduate college students	Eight-week mindfulness based stress reduction course by a certified MBSR instructor.	26	6 week sham mindfulness Control group		The Test Anxiety Inventory
Jahani et al., Iran	2020	20	Secondary school	Mindfulness training for a period of 10 sessions	20	No intervention	One and a half months	The Test Anxiety Scale
Shaidi et al., Iran	2017	25	High school	The MBSR training interventions, eight weekly 90-min sessions	25	No intervention	3-month follow-up	The test anxiety scale
Lothes et al., USA	2019	32	College students	Eight week mindfulness practices	11	No intervention	6-month follow up	Test Anxiety Scale
Niss, USA	2012	53	High school	Brief mindfulness intervention	54	No intervention	No follow up	The State- Trait Anxiety Inventory
Cho et al., Japan	2016	12	University students	Participants trained by themselves for 6 days after they had taken one session of education for mindful for 7 sessions	12	A training cognitive reappraisal condition and non-training condition control group	No follow up mentioned	The Revised Test Anxiety
Carsley et al.	2018	97	Grade 8 students	Mindfulness art activity	96	Free draw/coloring activity	No follow up mentioned	State–Trait Anxiety Inventory (STAI)
Lothes et al., USA	2022	10	College students	Mindfulness practices for 5 weeks	10	Wait list control	No follow up mentioned	Test Anxiety Inventory (TAI)
Savoie, USA	2016	32	University students	The mindfulness intervention consisted of four sessions (two sessions a week for 2 weeks), with each session lasting 30 min/8 sessions	32	No intervention	No follow up mentioned	Test Anxiety Inventory (TAI)
Wenger et al., Switzerland	2022	25	University students	Short mindfulness intervention with daily individual practice of the 30-min for 8 weeks	25	The training program (short intervention: Neutral story to listen individual practice of the 30-min same neutral story listening, for 8 weeks without any interaction with the instructor)	No follow up	State–Trait Anxiety Inventory (STAI-Y)
Contreras et al., USA	2020	26	High school	Two weeks of mindful deep breathing training	26	No intervention	No follow up mentioned	Test Anxiety Scale (TAS)
Reinhardt, USA	2014	45	Middle school, 6th graders	Discussion based instruction focusing on mindfulness, deep breathing	45	Control group was supervised by a School Psychologist and instructed to read a book of their choice for 30 min		Test Anxiety Scale for Mathematics (TASM)
Morrell, USA	2018	29	Middle school students	Mandala drawing, guided mindfulness practices	14	In a neutral task (reading short stories).		State–Trait Anxiety Inventory for Children (STAIC-S)
Paterniti, USA	2007	24	College students	Mindfulness practices body scan, mindful breathing	24	Skills training, number of different study skills, including time management, memory techniques, note-taking skills and ways to anticipate test content	No follow up	The Test Anxiety Inventory
Franco et al., Spain	2010	31	First year high school	10 sessions mindfulness intervention program	30	No intervention	No follow up	Spanish Version of the State–Trait Anxiety Inventory (STAI)
Collins et al., USA	2019	53	Undergraduate college students	The mindfulness intervention, multimedia training,1 group sessions, 5 individual sessions, total 6 sessions	54	No intervention	No follow up	The Westside Test Anxiety Scale
Seidi, Iraq	2018	15	University students	8 sessions of mindfulness-based stress reduction program	15	No intervention	No follow up	Spielberger’s Test Anxiety
Arjunan, India	2016	44	Secondary school	The Brief Mindfulness-Based Stress Reduction Program	44	No intervention	No follow up	Test-Anxiety Scale for Secondary School Students (TASS)
Dodson, USA	2021	39	University students	Students practiced mindfulness strategies for 16 weeks	39	No intervention	No follow up	The Test Anxiety Inventory (TAI)

### Overall summary effect

The meta-analysis, employing a random-effects model, assessed the efficacy of mindfulness-based interventions (MBIs) compared to no-treatment control groups across 20 comparisons. The aggregated effect size was found to be −0.716 (SE = 0.340; 95% CI -1.383 to 0.049; *Z* = −2.11, *p* = 0.035). This effect size is indicative of a moderate to large negative effect, suggesting a substantial reduction in test anxiety due to MBIs. Notably, the analysis uncovered a high degree of heterogeneity among the studies (*I^2^* = 99.95%), with a *Q* statistic of 104,553.6 (*df* = 19, *p* < 0.0001), pointing to substantial variability in the effect sizes across studies. The estimated variance of the true effects (*T*^2^ = 2.1275), highlighting the diversity in intervention outcomes. The forest plot, illustrating the individual study contributions, underscores the direction and magnitude of these effects. Each study’s impact is represented proportionally, reflecting its relative weight in the overall effect size calculation. In conclusion, the meta-analysis provides an evidence for the effectiveness of MBIs in diminishing test anxiety. The majority of the included studies reported statistically significant effect sizes, affirming the practical significance of MBIs in this area. The negative sign of the effect size aligns with the anticipated outcome of reduced anxiety levels post-intervention.

### Publication bias

**Figure 2:**
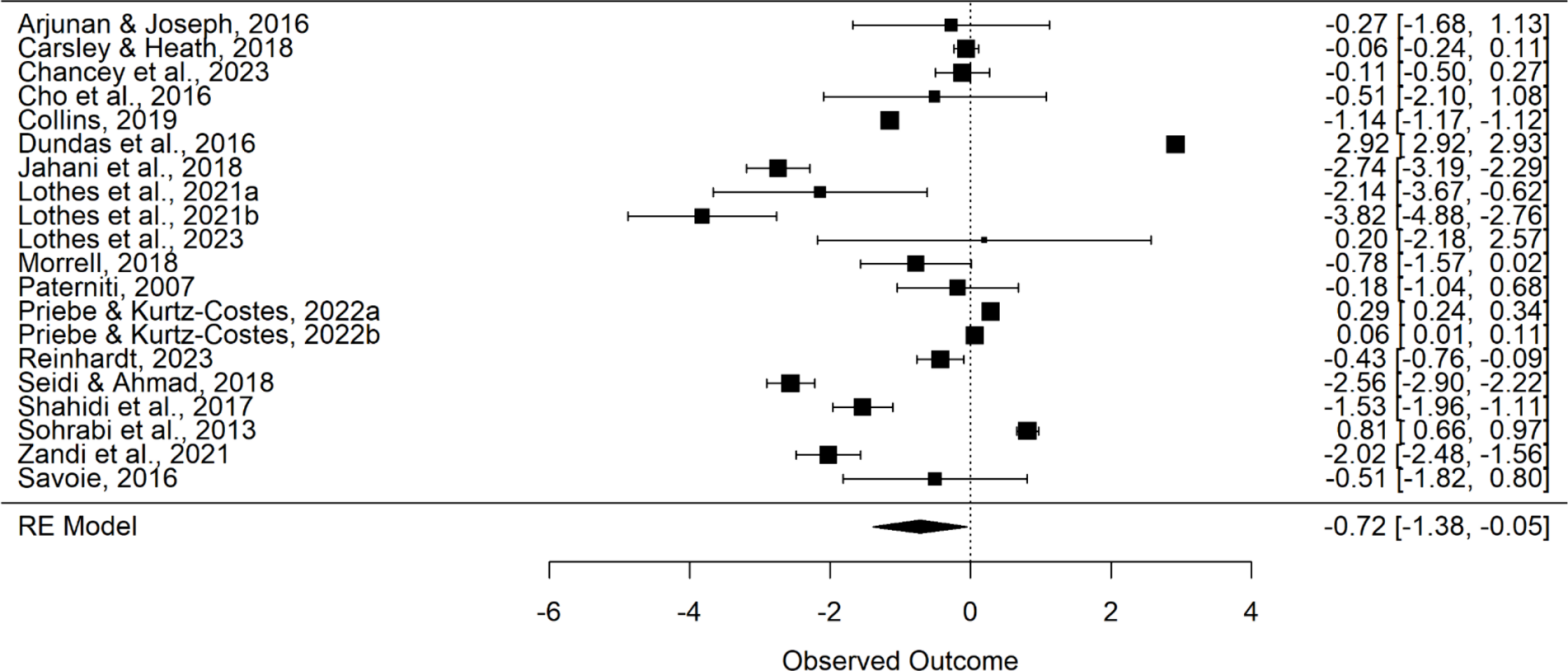
Forest plot—Cohen’s d.

Upon scrutiny of the funnel plot, asymmetry was observed, indicating potential publication bias. Egger’s regression revealed a significant intercept [*b* = 2.93, 95% CI: 2.74–3.13; *t* (18) = −2.44, *p* = 0.025], suggesting bias presence. In contrast, Begg and Mazumdar’s test did not signal publication bias (Kendall’s tau = 0.63, *p* < 0.0001). Duval and Tweedie’s trim and fill method detected no missing studies, implying no adjustment to the effect size was necessary. Due to these mixed findings, caution is advised in interpreting the meta-analytic results (Figure 2).

### Moderation analysis

**Figure 3:**
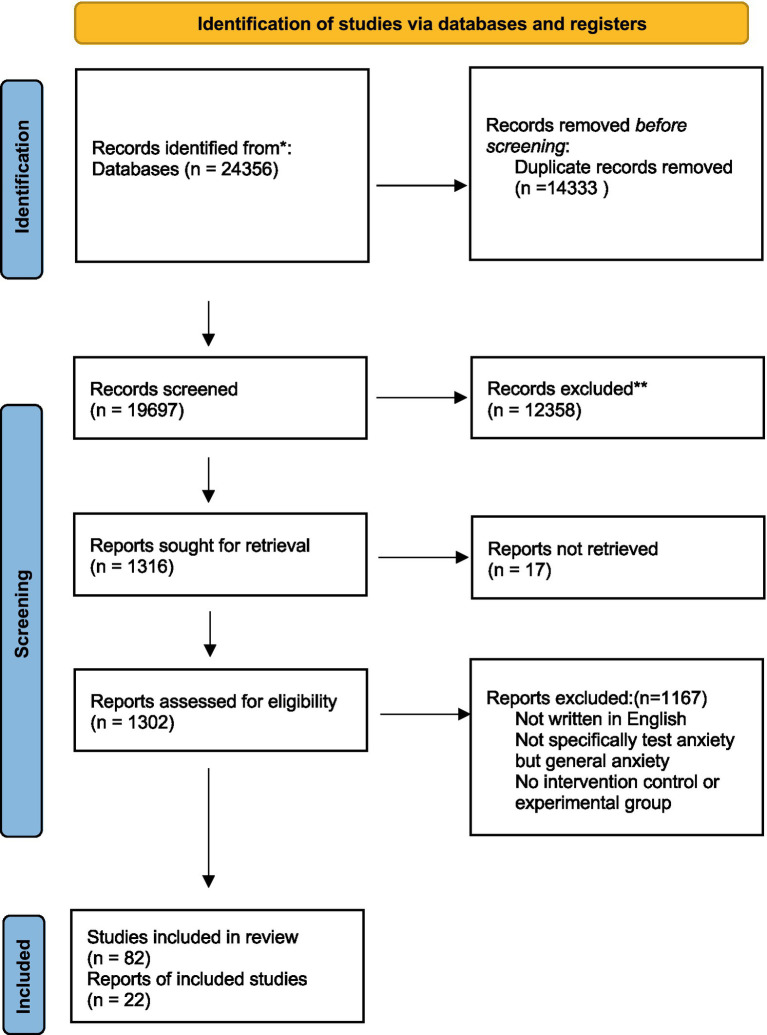
Forest plots of mindfulness interventions on test anxiety.

Moderation analyses were conducted to examine the influence of participant age, number of intervention sessions, and intervention delivery mode on the variability of effect sizes. The meta-regression approach was utilized, treating continuous moderators analogously to a multiple regression framework and categorical moderators through an analysis of variance equivalent. Mean participant age was not a significant moderator of effect sizes in the interventions (*b* = 0.04, SE = 0.08, *z* = 0.45, *p* = 0.65, 95% CI: −0.12 to 0.19), suggesting that the age of participants did not account for the observed heterogeneity in outcomes. Similarly, the number of sessions constituting the interventions did not significantly moderate the effect sizes (*b* = −0.05, SE = 0.03, *z* = −1.59, *p* = 0.11, 95% CI: −0.11 to 0.01), indicating that the frequency of sessions was not a determinant of intervention efficacy within the studies considered. The mode of intervention delivery, categorized as online versus face-to-face, was also not a significant moderator (b = 0.054, SE = 0.82, *z* = 0.066, *p* = 0.95, 95% CI: −1.54 to 1.65), pointing to a similar effectiveness of the interventions regardless of delivery method. No substantial reduction in residual heterogeneity was achieved through the inclusion of these moderators, as indicated by high I^2^ values in excess of 99% for all models. Consequently, the significant heterogeneity among studies persists unexplained by these moderator variable (Figure 3).

## Discussion

Many meta- analysis have been conducted on the effects of mindfulness for anxiety disorder but not specifically for test anxiety. Centralized exams or exams required for specialization in a field are included in the education systems of all countries, and uncontrollable test anxiety negatively affects physiological, emotional and performance levels of all age groups. Considering the limited studies on test anxiety, which is an indispensable part of the lives of individuals at all age levels and the increasing anxiety levels of various groups as the exams approach, it is thought that there is a need for studies that reveal the effectiveness of mindfulness studies. Therefore, this meta-analysis is conducted for revealing the effect of mindfulness-based interventions on test anxiety.

Researches have shown that mindfulness can affect exam anxiety. Although there are some researches about the interventions of mindfulness on exam anxiety have conducted, no meta-analysis have examined the effects of mindfulness on test anxiety. This article is a meta-analysis of effects of mindfulness interventions on exam anxiety which is conducted with secondary, middle, college, high school and adolescent students sample. At the same time, a limited number of studies examining exam anxiety, which is extremely important for students, have been accessed. The majority of the included studies reported statistically significant effect sizes, affirming the practical significance of MBIs in this area. The negative sign of the effect size aligns with the anticipated outcome of reduced anxiety levels post-intervention.

Although there are many studies demonstrating the positive effect of mindfulness studies on general anxiety disorders (Spijkerman et al., 2016), common psychiatric disorders (Hedman-Lagerlöf et al., 2018), adolescents’ stress, depression, and anxiety in school settings (Fulambarkar et al., 2023), stress management in the general population (Zhang et al., 2024), cognitive performance, emotional problems, stress and coping, resilience in school environment (Zenner et al., 2014), there has been no meta-analysis conducted specifically on the effects of MBSR practices on exam anxiety. This study is important because it is the first study to show that mindfulness-based interventions are effective in reducing students’ test anxiety. According to this research results, MBIs demonstrating a moderate to large negative effect on test anxiety. While supporting the conclusion, we inform that the findings related to publication bias should be taken into account when evaluating the results. Although Duval and Tweedie’s crop and fill method has not detected any missing studies, it is recommended to be careful in interpreting the meta-analytical results. Fulambarkar et al. (2023) conducted a met-analysis of mindfulness studies on adolescents’ stress, depression and anxiety levels in the school setting. As a result of the meta-analysis, the overall effect including stress, depression, and anxiety resulted in a significant improvement with a small effect size.

In a meta-analysis of mindfulness intervention on general anxiety of college students (Bamber and Morpeth, 2019), a certain high effect value was found in the group in which mindfulness meditation was applied compared to the control group. Although there is a meta-analysis on the effect of mindfulness interventions on general anxiety rather than exam anxiety, it was found important to be conducted on student population in order to compare the results.

Another meta-analysis on the effect of mindfulness interventions on the psychological health of children and adolescents (Kallapiran et al., 2015) includes 11 studies and according to the results, it was stated that the psychological health and life satisfaction of children and adolescents in the group with mindfulness intervention increased.

In prior meta-analyses, researchers studied the effects of MBIs on psychological distress and well-being, and attrition rates in university students and adolescents’ stress, depression, and anxiety in school settings (Fulambarkar et al., 2023; Alrashdi et al., 2024). There are other meta-analysis about mindfulness intervention effects on anxiety disorders, and anxiety symptoms in clinical and non-clinical samples (Zhihong et al., 2018; Fumero et al., 2020; Reangsing et al., 2023; Williams et al., 2023). Researchers reported that MBIs significantly reduced anxiety in both clinical and non- clinical samples in the youth population and reduced overall anxiety in adult clinical and non-clinical populations.

When the findings of our study were examined, it was found that mindfulness practices had a positive effect on reducing exam anxiety to a reasonable level. When the control and experimental groups of the studies included in the study were compared, it was found that mindfulness practices had a significant effect on reducing test anxiety of students at various levels. Each study’s impact is represented proportionally, reflecting its relative weight in the overall effect size calculation. In conclusion, the meta-analysis provides an evidence for the effectiveness of MBIs in diminishing test anxiety.

In the literature, there is no study examining the effects of MBIs on test anxiety. This study is considered to be the first meta-analysis of school-based MBIs on test anxiety in the academic field. It provides supportive evidence that students from various levels had reduced test anxiety after MBIs. There is a previous meta-analysis that investigated the effect of MBIs on the general anxiety of university students (Bamber and Morpeth, 2019) and the general ES findings of this study are consistent with the results of our meta-analysis study. Another review and meta-analysis is a school-based mindfulness study (Zenner et al., 2014), which included 19 studies and 1,348 students from Grade 1 to Grade 12. The findings of this meta-analysis are similar to ours. It was found that MBIs contributed positively to the psychological well-being of children and youth. And as similar to our study, heterogeneity calculated as high.

When the moderator variables in our study were examined (age, number of sessions, frequency of sessions and type of intervention), it was found that they did not mediate the effect level of mindfulness-based interventions. It is an important result that the studies conducted in a wide age range from middle school students to university students did not show a significant difference at the age level. While mindfulness-based interventions conducted with children and adolescents had significant effects in children but not in adolescents (Odgers and Jensen, 2020), other studies found that mindfulness interventions conducted with children and adolescents did not have any effect according to age (Zoogman et al., 2015; Kander et al., 2024). On the contrary, in other studies, age was found to be an important moderator (Dunning et al., 2019; Mettler et al., 2023). However, it is useful to emphasize at this point. Although these studies were based on mindfulness, they did not specifically examine its effects on test anxiety and investigated its effects on different variables such as mental health, wellbeing, psychological symptoms, academic achievement, impulsivity, and interpersonal relationships. Looking at the results of the meta-analysis, it can be stated that there is a need for a comprehensive research that reveals the effects of developmental levels as an important moderator. In addition, it should be noted that this study did not include children, but included an age range from middle school to university students and the average age was 18.93.

In this study, it was also observed that other moderator variables such as the number and frequency of sessions and the type of intervention (online or face-to-face) did not have a significant mediating effect of mindfulness-based interventions on test anxiety. This finding is consistent with some of the previous meta-analytic studies on the effects of mindfulness-based interventions (Zoogman et al., 2015; Kander et al., 2024). Previously, in a meta-analysis conducted with college students examining the effect of mindfulness on general anxiety, it was shown that the face-to-face and long-term mindfulness studies of MBIs increased the effectiveness (Bamber and Morpeth, 2019). However, in our study, we observed that these differences in practice did not reveal a significant effect. This may be the result of MBIs worked on general anxiety. This made us think that MBI is more effective in more specific problems and groups. We can say that MBIs are more effective in a specific state anxiety such as exam anxiety.

The findings of the study showed that mindfulness interventions reduced test anxiety in student groups at various levels, but since one of the main problems of meta-analysis studies is publication bias, it is necessary to consider the possibility of publication bias.

Implementing mindfulness-based interventions in schools helps students learn efficiently with mindfulness and then demonstrate what they have learned by controlling their anxiety. These interventions can also be beneficial for both students and teachers’ psychological resilience, psychological well-being, self-esteem and prevention of burn out.

## Limitations

One limitation in this meta-analysis is the small number of studies that met our inclusion criteria. Additionally, there was significant heterogeneity among studies.

## Data availability statement

The original contributions presented in the study are included in the article/supplementary material, further inquiries can be directed to the corresponding author.

## Author contributions

EY: Data curation, Methodology, Writing – original draft, Writing – review & editing. ZH: Supervision, Writing – review & editing. FT: Supervision, Validation, Writing – review & editing.

## References

[ref1] Alrashdi D. H. Chen K. K. Meyer C. Gould R. L. (2024). A systematic review and meta-analysis of online mindfulness-based interventions for university students: an examination of psychological distress and well-being, and attrition rates. J. Technol. Behav. Sci. 9, 211–223. doi: 10.1007/s41347-023-00321-6

[ref2] Bamber M. D. Morpeth E. (2019). Effects of mindfulness meditation on college student anxiety: a meta-analysis. Mindfulness 10, 203–214. doi: 10.1007/s12671-018-0965-5, PMID: 24176784

[ref3] Bartlett L. Buscot M. J. Bindoff A. Chambers R. Hassed C. (2021). Mindfulness is associated with lower stress and higher work engagement in a large sample of MOOC participants. Front. Psychol. 12:724126. doi: 10.3389/fpsyg.2021.724126 34566805 PMC8461060

[ref4] Bishop S. R. Lau M. Shapiro S. . (2004). Mindfulness: a proposed operational definition. Clin. Psychol. Sci. Pract. 11, 230–241. doi: 10.1093/clipsy.bph077, PMID: 37795325

[ref5] Breedvelt J. Amanvermez Y. Harrer M. Karyotaki E. Gilbody S. Bockting C. . (2019). The effects of meditation, yoga, and mindfulness on depression, anxiety, and stress in tertiary education students: a meta-analysis. Front. Psych. 10:193. doi: 10.3389/fpsyt.2019.00193 PMC6491852 31068842

[ref6] Cardaciotto L. Herbert J. D. Forman E. M. Moitra E. Farrow V. (2008). The assessment of present-moment awareness and acceptance: the Philadelphia mindfulness scale. Assessment 15, 204–223. doi: 10.1177/1073191107311467, PMID: 18187399

[ref01] Chiesa A. Serretti A. (2010). Mindfulness based cognitive therapy for psychiatric disorders: a systematic review and meta-analysis. Psychiatry Res. 187, 441–453. doi: 10.1016/j.psychres.2010.08.011 20846726

[ref7] D’Agostino A. Schirripa Spagnolo F. Salvati N. (2022). Studying the relationship between anxiety and school achievement: evidence from PISA data. Stat. Methods Appl. 31, 1–20. doi: 10.1007/s10260-021-00563-9

[ref8] Dunning D. L. Griffiths K. Kuyken W. Crane C. Foulkes L. Parker J. . (2019). Research review: the effects of mindfulness-based interventions on cognition and mental health in children and adolescents - a meta-analysis of randomized controlled trials. J. Child Psychol. Psychiatry 60, 244–258. doi: 10.1111/jcpp.12980, PMID: 30345511 PMC6546608

[ref9] Edenfield T. M. Saeed S. A. (2012). An update on mindfulness meditation as a self-help treatment for anxiety and depression. Psychol. Res. Behav. Manag. 5, 131–41. doi: 10.2147/PRBM.S34937 23175619 PMC3500142

[ref10] Ergene T. (2003). Effective interventions on test anxiety reduction: a meta-analysis. Sch. Psychol. Int. 24, 313–328. doi: 10.1177/01430343030243004, PMID: 38727072

[ref11] Eysenck M. W. Derakshan N. Santos R. Calvo M. G. (2007). Anxiety and cognitive performance: attentional control theory. Emotion 7, 336–353. doi: 10.1037/1528-3542.7.2.336, PMID: 17516812

[ref12] Fulambarkar N. Seo B. Testerman A. Rees M. Bausback K. Bunge E. (2023). Review: meta-analysis on mindfulness-based interventions for adolescents’ stress, depression, and anxiety in school settings: a cautionary tale. Child Adolesc. Ment. Health. 28, 307–317. doi: 10.1111/camh.12572, PMID: 35765773

[ref13] Fumero A. Peñate W. Oyanadel C. Porter B. (2020). The effectiveness of mindfulness-based interventions on anxiety disorders. A systematic meta-review. Eur. J. Investig. Health Psychol. Educ. 10, 704–719. doi: 10.3390/ejihpe10030052, PMID: 34542506 PMC8314302

[ref14] Goldin P. R. Gross J. J. (2010). Effects of mindfulness-based stress reduction (MBSR) on emotion regulation in social anxiety disorder. Emotion 10, 83–91. doi: 10.1037/a0018441, PMID: 20141305 PMC4203918

[ref15] Hedman-Lagerlöf M. Hedman-Lagerlöf E. Öst L. (2018). The empirical support for mindfulness-based interventions for common psychiatric disorders: a systematic review and meta-analysis. Psychol. Med. 48, 2116–2129. doi: 10.1017/s0033291718000259 29455695

[ref16] Hembree R. (1988). Correlates, causes, effects, and treatment of test anxiety. Rev. Educ. Res. 58, 47–77. doi: 10.3102/00346543058001047, PMID: 38840215

[ref17] Hernández-Saca D. I. (2016). Re-framing the master narratives of dis/ability through an emotion lens: voices of Latina/o students with learning disabilities (doctoral dissertation). Tempe: Arizona State University.

[ref18] Hölzel B. K. Carmody J. Vangel M. Congleton C. Yerramsetti S. M. Gard T. . (2011). Mindfulness practice leads to increases in regional brain gray matter density. Psychiatry Res. 191, 36–43. doi: 10.1016/j.pscychresns.2010.08.006 21071182 PMC3004979

[ref19] Hughes B. M. (2005). Study, examinations, and stress: blood pressure assessments in college students. Educ. Rev. 57, 21–36. doi: 10.1080/0013191042000274169, PMID: 38454093

[ref20] Kabat-Zinn J. (2003). Mindfulness-based interventions in context: past, present, and future. Clin. Psychol. Sci. Pract. 10, 144–156. doi: 10.1093/clipsy.bpg016

[ref21] Kallapiran K. Koo S. Kirubakaran R. Hancock K. (2015). Effectiveness of mindfulness in improving mental health symptoms of children and adolescents: a meta-analysis. Child. Adolesc. Ment. Health 20, 182–194. doi: 10.1111/camh.12113 32680348

[ref22] Kander T. N. Lawrence D. Fox A. Houghton S. Becerra R. (2024). Mindfulness-based interventions for preadolescent children: a comprehensive meta-analysis. J. Sch. Psychol. 102:101261. doi: 10.1016/j.jsp.2023.101261, PMID: 38143094

[ref23] Kiken L. G. Lundberg K. B. Fredrickson B. L. (2017). Being present and enjoying it: dispositional mindfulness and savoring the moment are distinct, interactive predictors of positive emotions and psychological health. Mindfulness (NY) 8, 1280–1290. doi: 10.1007/s12671-017-0704-3 PMC5755604 29312472

[ref24] Kuyken W. Watkins E. Holden E. White K. Taylor R. S. Byford S. . (2010). How does mindfulness-based cognitive therapy work? Behav. Res. Ther. 48, 1105–1112. doi: 10.1016/j.brat.2010.08.003, PMID: 20810101

[ref25] Linehan M. M. (1993). Cognitive-behavioral treatment of borderline personality disorder. New York, NY: Guilford Press.

[ref26] Linsay S. (2002). The effect of test anxiety on attention and memory skills in undergraduate students. Paper presented at the Annual Review of undergraduate research at the College of Charleston, USA Charleston.

[ref27] Lutz A. Brefczynski-Lewis J. Johnstone T. Davidson R. J. (2008a). Regulation of neural circuitry of emotion by compassion meditation: effects of meditative expertise. PLoS One 3:e1897. doi: 10.1371/journal.pone.0001897, PMID: 18365029 PMC2267490

[ref28] Lutz A. Slagter H. A. Dunne J. Davidson R. J. (2008b). Attention regulation and monitoring in meditation. Trends Cogn. Sci. 12, 163–169. 18329323 10.1016/j.tics.2008.01.005 PMC2693206

[ref29] Malinowski P. (2013). Neural mechanisms of attentional control in mindfulness meditation. Front. Neurosci. 7:8. doi: 10.3389/fnins.2013.00008 23382709 PMC3563089

[ref30] Maran T. Woznica M. Moder S. Furtner M. Jehle E. Hörner S. . (2021). Correction to: overcoming automaticity through meditation. Mindfulness 12:3109. doi: 10.1007/s12671-021-01776-5

[ref31] Mettler J. Khoury B. Zito S. Sadowski I. Heath N. L. (2023). Mindfulness-based programs and school adjustment: a systematic review and meta-analysis. J. Sch. Psychol. 97, 43–62. doi: 10.1016/j.jsp.2022.10.007, PMID: 36914366

[ref32] Odgers C. L. Jensen M. R. (2020). Annual research review: adolescent mental health in the digital age: facts, fears, and future directions. J. Child Psychol. Psychiatry 61, 336–348. doi: 10.1111/jcpp.13190, PMID: 31951670 PMC8221420

[ref33] OECD (2017). “Schoolwork-related anxiety” in PISA 2015 results (volume III): students' well-being (Paris: OECD Publishing).

[ref34] Purser R. Loy D. (2013). Beyond McMindfulness. Huffington. Available at: http://www.huffingtonpost.com/ron-purser/beyond-mcmindfulness_b_3519289.html (Accessed February 21, 2014).

[ref35] Rapgay L. Bystrisky A. (2009). Classical mindfulness: an introduction to its theory and practice for clinical application. Ann. N. Y. Acad. Sci., 1172, 148–162. doi: 10. 1111/j.1749–6632.2009.04405.x, doi: 10.1111/j.1749-6632.2009.04405.x, PMID: 19735247

[ref36] Reangsing C. Trakooltorwong P. Maneekunwong K. Thepsaw J. Oerther S. (2023). Effects of online mindfulness-based interventions (MBIs) on anxiety symptoms in adults: a systematic review and meta-analysis. BMC Complement Med Ther. 23:269. doi: 10.1186/s12906-023-04102-9 37507747 PMC10386675

[ref37] Segal Z. Williams J. Teasdale J. (2012). Mindfulness-based cognitive therapy for depression. 2nd Edn. New York: Guilford Press.

[ref38] Spijkerman M. P. J. Pots W. T. M. Bohlmeijer E. T. (2016). Effectiveness of online mindfulness-based interventions in improving mental health: a review and meta-analysis of randomised controlled trials. Clin. Psychol. Rev. 45, 102–114. doi: 10.1016/j.cpr.2016.03.009, PMID: 27111302

[ref39] Taren A. A. Creswell J. D. Gianaros P. J. (2013). Dispositional mindfulness co-varies with smaller amygdala and caudate Vols. In community adults. PLoS One 8:e64574. doi: 10.1371/journal.pone.0064574 23717632 PMC3661490

[ref40] Von der Embse N. Barterian J. Segool N. (2013). Test anxiety interventions for children and adolescents: a systematic review of treatment studies from 2000–2010. Psychol. Sch. 50, 57–71. doi: 10.1002/pits.21660

[ref41] Von der Embse N. Jester D. Roy D. Post J. (2018). Test anxiety effects, predictors, and correlates: a 30-year meta-analytic review. J. Affect. Disord. 227, 483–493. doi: 10.1016/j.jad.2017.11.048, PMID: 29156362

[ref42] Williams M. Honan C. Skromanis S. Sanderson B. Matthews A. J. (2023). Psychological outcomes and mechanisms of mindfulness-based training for generalised anxiety disorder: a systematic review and meta-analysis. Curr. Psychol. 11, 1–23. doi: 10.1007/s12144-023-04695-x PMC10173921 37359641

[ref43] Zeidner M. (1998). Test anxiety: the state of the art. New York: Plenum Press.

[ref44] Zenner C. Herrnleben-Kurz S. Walach H. (2014). Mindfulness-based interventions in schools—a systematic review and meta-analysis. Front. Psychol. 5:603. doi: 10.3389/fpsyg.2014.00603, PMID: 25071620 PMC4075476

[ref45] Zhang Z. Hu Y. Liu S. Feng X. Yang J. Cheng L. J. . (2024). The effectiveness of e-mental health interventions on stress, anxiety, and depression among healthcare professionals: a systematic review and meta-analysis. Syst. Rev. 13:144. doi: 10.1186/s13643-024-02565-6 38816879 PMC11138032

[ref02] Zhihong R. Yawen Z. Guangrong J. (2018). Effectiveness of mindfulness meditation in intervention for anxiety: a meta-analysis. Acta Psychologica Sinica 3, 283–305. doi: 10.3724/SP.J.1041.2018.00283

[ref46] Zoogman S. Goldberg S. B. Hoyt W. T. Miller L. (2015). Mindfulness interventions with youth: a meta-analysis. Mindfulness 6, 290–302. doi: 10.1007/s12671-013-0260-4, PMID: 38722532

